# Coronavirus Disease 2019 (COVID-19): Considerations for the Competitive Athlete

**DOI:** 10.1177/1941738120918876

**Published:** 2020-04-06

**Authors:** Brett G. Toresdahl, Irfan M. Asif

**Affiliations:** † Department of Primary Care Sports Medicine, Hospital for Special Surgery, New York, New York; ‡ Department of Family and Community Medicine, University of Alabama at Birmingham, Birmingham, Alabama

In late 2019, a previously unidentified novel strain of coronavirus was identified as the cause of several cases of pneumonia in Wuhan, China. “Coronavirus disease 2019” (COVID-19), declared a pandemic by the World Health Organization (WHO), has been responsible for hundreds of thousands of cases worldwide, with sustained or widespread transmission initially occurring in China, South Korea, Iran, Italy, and Japan, then spreading to the majority of Europe and the United States. COVID-19 enters the body through the same cell receptors as severe acute respiratory syndrome (SARS) and is distantly related to Middle East respiratory syndrome (MERS).^15^ Bats appear to be the primary source, given the similarity in RNA sequencing to 2 bat coronaviruses.^15^ Typical features of the illness include fever, fatigue, cough, and myalgias.

Although athletes are younger and have fewer comorbidities than the general population, and therefore are at lower risk for severe disease or death,^27^ preventing the transmission of COVID-19 is necessary to protect those at high risk of death and to slow the pandemic so that health care systems do not exceed their capacities. Sports medicine providers involved in the care of competitive athletes should be aware of the prevention strategies for COVID-19, common symptoms for the disease, potential treatment options, and when it may be safe to return to athletic participation after infection.

## COVID-19 Impact on Sports

All major sports leagues and tournaments have been suspended or canceled due to COVID-19 since early March 2020. Initially, some sporting events were to be held without spectators to reduce transmission through close contact among fans.^12,13^ In the case of the National Basketball Association, the season was suspended soon after a player tested positive for COVID-19.^16^ Other sporting events were forced to cancel when local and state governments restricted the sizes of gatherings.^3^ On March 24, 2020, the International Olympic Committee announced that the Olympic and Paralympic Games Tokyo 2020 would be postponed to Summer 2021.^14^

## Prevention of COVID-19 in Athletes

### Purpose of Prevention

While the typical athlete may only experience mild symptoms as a result of COVID-19, prevention strategies are necessary for multiple reasons. First and foremost, preventing the transmission of COVID-19 is needed to reduce the risk of spread to individuals within a community who are most at risk of severe infection or death, which includes older individuals and the immunocompromised.^27^ Prevention of COVID-19 is also important for the competitive athlete to minimize interruptions in training and the adverse effects that it could have on his or her respiratory tract and aerobic capacity in both the short and long term.

### Preventing Transmission

While the first cases of COVID-19 were associated with a seafood market in Wuhan, the virus has since spread person-to-person primarily via respiratory droplets.^15,26^ This mode of transmission occurs when the virus, in the form of respiratory secretions from coughing or sneezing, contacts another person’s mucous membranes. According to Chinese data, the rate of secondary COVID-19 infections ranges from 1% to 5%.^26^ Transmission can also occur if a person touches his or her eyes, nose, or mouth after touching a surface containing respiratory droplets with the virus, which can remain viable for hours to days.^7^ Presymptomatic/asymptomatic carriers, which comprised 48% of the 531 cases on the *Diamond Princess* cruise ship, are also capable of transmitting COVID-19.^2,17,28^ Currently, there is no evidence that the virus is spread through the shipment of food or other products from overseas.

Sports medicine providers can support athletes and teams during the COVID-19 pandemic by advocating the following preventative measures:

*Hand hygiene*: General guidelines include washing hands often with soap and water for at least 20 seconds or using hand sanitizer (at least 60% alcohol) if soap and water are not available. As the virus can survive for days on surfaces, frequently touched objects and surfaces should be regularly cleaned and disinfected.^22^
*Social distancing*: The Centers for Disease Control and Prevention (CDC) describes social distancing as remaining out of congregate settings, avoiding mass gatherings, and maintaining distance (approximately 6 feet) from others when possible.^9^ This practice is being advocated by governments and promoted by professional athletes as well.^4,19^
*Travel*: To slow transmission, many countries have imposed travel restrictions. Measures have ranged from suspending flights, to banning travelers from affected countries, to in-home isolation for 14 days after returning from specific destinations. Countries are also performing entry screening, including measuring body temperature and assessing for signs and symptoms of COVID-19. Domestic travel has become challenging as busy airports can be a common site of person-to-person spread. However, as a result of the sweeping suspensions and cancelations of sports leagues and tournaments, many athletes are not needing to travel beyond returning home from where they were training or competing.
*Face mask*: Asymptomatic athletes should not be advised to wear a mask to prevent becoming infected with COVID-19 in the community setting or while traveling since it does not significantly reduce the risk of infection.^8^ Inappropriate use of masks can affect supply and demand to the point where health care workers will have inadequate protection, as we are currently seeing.

### Training Modification

Prolonged and strenuous training has been suggested to be associated with temporary immune system depression lasting hours to days.^21^ A conservative approach would be to advise athletes to limit training sessions to <60 minutes and to <80% of maximum ability during this time to prevent COVID-19. However, this “open window” theory of infection susceptibility that follows a bout of vigorous exercise has been challenged.^5^

### Immunization

Vaccines are in the early stages of development but are unlikely to be available until early to mid-2021.

## Symptoms of COVID-19 Infection

The incubation period is typically within 14 days from exposure, with 95% of cases occurring within 5 days. The most common symptoms include fever (99%), fatigue (70%), dry cough (59%), and myalgias (35%).^23^ Some may also experience anosmia (loss of smell), dysgeusia (altered taste), a sore throat, rhinorrhea, or gastrointestinal manifestations. Pneumonia is the most common serious manifestation, with bilateral infiltrates seen on chest imaging. Of nearly 50,000 cases in China, 81% were mild (did not require hospitalization), 14% were severe (dyspnea, hypoxia, or >50% lung involvement on imaging within 24-48 hours), and 5% were critical (respiratory failure, shock, or organ failure).

Influenza and bacterial pneumonia should be considered when evaluating an athlete with fever, cough, and/or shortness of breath. Testing for influenza can be done either prior to testing for COVID-19 or simultaneously. A complete blood count to look for leukocytosis can help determine whether the symptoms are caused by a bacterial pneumonia. Conversely, lymphopenia and leukopenia have been seen in COVID-19 infections, which may assist in diagnosis.^2^

## Testing Athletes with Suspected COVID-19

During the early course of the spread of COVID-19, availability of outpatient testing for the virus has lagged behind clinical needs. With these limitations, testing algorithms offered preference to patients with symptoms (fever, cough, or shortness of breath), an immunocompromised state, or close contact with someone with COVID-19. As more tests are developed and approved in the United States, including those with faster turnaround times, testing criteria are expected to expand and may include testing asymptomatic individuals, as was done in South Korea.^11^

Testing is done with a nasopharyngeal swab using an RNA detection polymerase chain reaction (PCR) test. Retesting may be needed in those with a negative initial test and a high probability of disease. A chest computed tomography scan can also be used to evaluate for signs of viral pneumonia as reverse transcription PCR may not detect COVID-19 early in the course of the infection.^1^

## Management of an Athlete with COVID-19

The management of COVID-19 infection depends on the severity of symptoms. In New York City, 10% of individuals age 18-45 who tested positive for COVID-19 required hospitalization.^18^ However, given the limited access to testing and variable symptomatology, the total number of individuals with COVID-19 may be much higher so the true risk of hospitalization among this age group is likely lower. Therefore, for an otherwise healthy athlete under age 45 who becomes infected with COVID-19, he or she would likely experience a self-limited flu-like illness. Managing symptoms in an athlete primarily involves symptomatic management with rest and over-the-counter antipyretics.

### In-Home Isolation

In-home isolation is recommended for athletes with confirmed or suspected COVID-19 who do not show severe symptoms. Other members of the household should minimize time in the same room as the affected individual, who should wear a mask when others are present.

### Antipyretics

The health minister of France recently advocated for use of acetaminophen to treat fever associated with COVID-19 and suggested that ibuprofen could worsen the infection.^24^ This appeared to be based on a theoretical concern that the anti-inflammatory effects of nonsteroidal anti-inflammatory drugs (NSAIDs) could adversely affect the immune system. However, the WHO currently does not recommend against using NSAIDs when clinically indicated in the treatment of a COVID-19 infection.

### Corticosteroids

The WHO recommends that corticosteroids not be used in patients with COVID-19 pneumonia unless there are other indications, such as the exacerbation of chronic obstructive pulmonary disease.^25^ Corticosteroids have been associated with an increased risk for mortality in patients with influenza and delayed viral clearance in patients with MERS. There has also been good evidence for short- and long-term harm in SARS patients treated with corticosteroids.^20^

### Drugs Under Investigation

The following agents are being investigated as potential treatment options. It is important to note that there are currently no controlled data supporting the use of these medications and their efficacy is unknown.

*Remdesivir*: Randomized clinical trials are under way assessing this investigational antiviral nucleotide analog in hospitalized adults. It has shown promise in in vitro as well as in animal studies.
*Lopinavir-ritonavir*: There have been case reports of treatment with this protease inhibitor used in HIV treatment, which has shown in vitro activity against MERS and SARS. However, 1 trial of nearly 200 patients with severe COVID-19 infection showed no difference in time to symptom resolution or mortality when compared with standard supportive treatment.^6^
*Hydroxychloroquine/chloroquine*: Studies are ongoing to investigate these 2 agents, which have shown activity against COVID-19 in vitro. Hydroxychloroquine may have more potent antiviral activity. Published clinical data are limited, and caution should be used given potential side effects, such as QT prolongation.

### Discontinuation of In-Home Isolation

The CDC recommends discontinuing home isolation using either a test-based strategy or non–test-based strategy, depending on availability of testing resources.^10^ If a test-based strategy is used, home isolation can be discontinued when the following criteria are all met:

- No fever is present without the use of fever-reducing medications
- Resolution of respiratory symptoms
- Two consecutive negative COVID-19 tests collected ≥24 hours apart

When a non–test based strategy is used, the following criteria must be met:

- At least 7 days have passed since the appearance of symptoms
- At least 72 hours (3 days) have passed since recovery of symptoms without the use of fever-reducing medications

### Mental Health Support

Suspending seasons and canceling competitions can cause significant grief, stress, anxiety, frustration, and sadness for an athlete. The psychological impact of COVID-19 on a competitive athlete is potentiated by the removal of his or her social support network and normal training routine, which for some is a critical component of managing depression or anxiety. Sports medicine providers should anticipate the need for additional mental health support for athletes, which could include ensuring regular check-ins with athletes, facilitating telehealth consultation with a sports psychologist, and encouraging maintenance of social interactions with family, friends, and teammates by phone or video chat.

## Management of a Sports Team with COVID-19

If an athlete on a sports team develops symptoms consistent with COVID-19, teammates, coaches, and other staff who had close contact with the athlete (within 6 feet) in the preceding 14 days should begin in-home isolation. If the athlete undergoes testing, contacts can discontinue isolation if the test result is negative for COVID-19. However, if the test result is positive for COVID-19 (or if testing is not pursued and the athlete is treated presumptively), close contacts will need to continue their in-home isolation for 14 days from the last contact with the athlete. There will likely be requests for testing from asymptomatic teammates, coaches, and other staff. Testing availability will likely dictate whether these individuals can be tested. During this time, any symptoms experienced by other athletes or staff should be reported to the team physician to determine whether they are legitimate signs of COVID-19. Team physicians may also consider implementing daily temperature checks.

## Return to Training

For athletes with confirmed or presumed COVID-19, training can begin once symptoms completely resolve and energy levels return to normal. Since in-home isolation is necessary for at least 72 hours after resolution of symptoms, low-intensity indoor training may be attempted during that time. After discontinuing in-home isolation, an athlete can gradually return to training as tolerated. For asymptomatic athletes who are isolated due to recent travel or close contact with an individual with COVID-19, maintaining cardiovascular fitness may be difficult. Exercise that is recommended during the in-home isolation period is dependent on the available equipment, which may include a stationary bike, treadmill, and resistance training. Guidance and monitoring by a strength and conditioning coach or exercise physiologist can be provided remotely.

## Conclusion

As of March 2020, COVID-19 has become a global pandemic, halting athletic competition worldwide. Current focus is on the prevention of viral spread through social distancing and other common hygiene measures. Sports medicine providers should know the most common symptoms of COVID-19, work within their environments to learn and develop testing protocols as indicated by local resources, and minimize spread among teams. Treatment in the outpatient setting is mainly supportive and includes home isolation, although several treatment drugs are under clinical investigation.
